# 3D Printing Direct Powder Extrusion in the Production of Drug Delivery Systems: State of the Art and Future Perspectives

**DOI:** 10.3390/pharmaceutics16040437

**Published:** 2024-03-22

**Authors:** Ángela Aguilar-de-Leyva, Marta Casas, Carmen Ferrero, Vicente Linares, Isidoro Caraballo

**Affiliations:** Department of Pharmacy and Pharmaceutical Technology, Faculty of Pharmacy, Universidad de Sevilla, 41012 Sevilla, Spain; aguilardeleyva@us.es (Á.A.-d.-L.); cferrero@us.es (C.F.) vlinares@us.es (V.L.); caraballo@us.es (I.C.)

**Keywords:** 3D printing, direct powder extrusion, rheological properties, drug delivery systems, personalized medicine

## Abstract

The production of tailored, on-demand drug delivery systems has gained attention in pharmaceutical development over the last few years, thanks to the application of 3D printing technology in the pharmaceutical field. Recently, direct powder extrusion (DPE) has emerged among the extrusion-based additive manufacturing techniques. It is a one-step procedure that allows the direct processing of powdered formulations. The aim of this systematic literature review is to analyze the production of drug delivery systems using DPE. A total of 27 articles have been identified through scientific databases (Scopus, PubMed, and ScienceDirect). The main characteristics of the three types of 3D printers based on DPE have been discussed. The selection of polymers and auxiliary excipients, as well as the flowability of the powder mixture, the rheological properties of the molten material, and the printing temperatures have been identified as the main critical parameters for successful printing. A wide range of drug delivery systems with varied geometries and different drug release profiles intended for oral, buccal, parenteral, and transdermal routes have been produced. The ability of this technique to manufacture personalized, on-demand drug delivery systems has been proven. For all these reasons, its implementation in hospital settings in the near future seems promising.

## 1. Introduction

Three-dimensional printing (3DP) has been introduced as an innovative and versatile technology to manufacture drug delivery systems due to its precision and accuracy, efficiency, personalized prescription, and ability to create complex and customized dosage forms. Extrusion-, powder-, and laser-based techniques are the main categories of 3DP techniques, with fused deposition modeling (FDM) and semisolid extrusion (SSE) being the most commonly used extrusion-based techniques in pharmaceutical development. FDM involves the extrusion of a filament obtained from a mixture of thermoplastic polymers, drugs, and/or auxiliary excipients. It allows the production of solid dosage forms with customized doses [1], release profiles [2], or geometries [3,4], and a combination of different drugs [5,6], which demonstrates its efficacy in the context of personalized medicine [7]. It can also be highlighted by the cost-efficiency and wide range of printers and materials used. However, FDM presents several limitations, such as the need for filaments (usually obtained by hot melt extrusion (HME)) with appropriate mechanical and physical properties (homogeneous diameter and adequate stiffness and brittleness), the double heating and consequent thermal stress of the pharmaceutical ingredients, and the tendency of amorphous drug molecules to recrystallize during storage [8]. For all the above reasons, the process of filament optimization is time-consuming, and there is a limitation in the choice of drugs and excipients.

SSE is an alternative technique based on the extrusion of highly viscous liquids or semisolid materials (gels, pastes, or solids with a relatively low melting point) from pre-filled syringes at room or moderate temperatures, making it more suitable for thermolabile drugs. However, a post-printing solidification process is often required. The complete solvent removal in some cases and the risk of material collapse and loss of shape are other drawbacks of this technique [9,10].

A new material extrusion 3DP technique called direct pellet extrusion (DPE) was introduced in 2017 as a promising alternative to FDM printing in the plastics industry [11]. This technique involved the extrusion of pellets or powders previously melted in a “melt generation unit” through the die of a single-screw extruder to the nozzle of the printer to be directly printed without the previous preparation of filaments using HME. 

This way, the equipment necessary to process the formulations is reduced [12]. In addition, mixtures that cannot be printed by FDM due to the inappropriate mechanical properties of the filaments obtained could be potentially extruded using this technology. In DPE, the material flow towards the printer nozzle is mainly driven by the screw rotation, which is slightly influenced by the material’s mechanical properties [13,14,15,16]. Moreover, the thermal stress experienced by the APIs processed by DPE is significantly reduced in comparison with FDM [17,18,19].

Additionally, unlike HME, which usually requires high amounts of excipients to avoid producing too brittle or too flexible filaments to be loaded into an FDM printer, this technique overcomes such a limitation [18], showing its ability to print systems with high drug loadings even without additional excipients [16].

Another important advantage of this technique is the small amount of powder mixture required, which is useful when a small quantity of a drug is available, as in the case of the preclinical studies [20]. 

The objective of this systematic review is to analyze the application of DPE techniques to the manufacture of drug delivery systems. An overview of the 3D DPE printers used, the critical parameters of the formulation and printing process, and the drug delivery systems produced with this technique is carried out. Finally, future perspectives for the implementation of this technology in healthcare centers are discussed. 

## 2. Methodology

This systematic research was carried out following the Preferred Reporting Items for Systematic Reviews and Meta-Analyses Statement criteria (PRISMA) [21]. Scopus, PubMed, and Science Direct databases were searched. In each database, the search was extended to all fields of the documents since sometimes DPE is called by other names such as hot-melt 3D extrusion in the title, keywords, and abstract. The following terms and Boolean operator were used: “[direct powder extrusion]” OR “[direct extrusion]”. In the Scopus database, the subject area was limited to “Pharmacology, Toxicology and Pharmaceutics”, “Medicine” and “Health professions” and the document type was limited to “article”. In the Science Direct database, the subject area was limited to “Pharmacology, Toxicology and Pharmaceutical Science” and the document type was limited to “research articles”. Duplicated documents were excluded. Despite restricting the search to research articles, manual screening had to be carried out later as some review articles were not filtered by the automated tool.

The eligibility of each article was based on an analysis of the title and the abstract. The main eligibility criterion was “the direct extrusion of pharmaceutical formulations based on granules or powders at a temperature higher than 100 °C”. After this initial phase, the full article was read in order to carry out systematic data collection. Articles not accessible or written in a language different from Spanish, English, or French were excluded.

**Figure 1:**
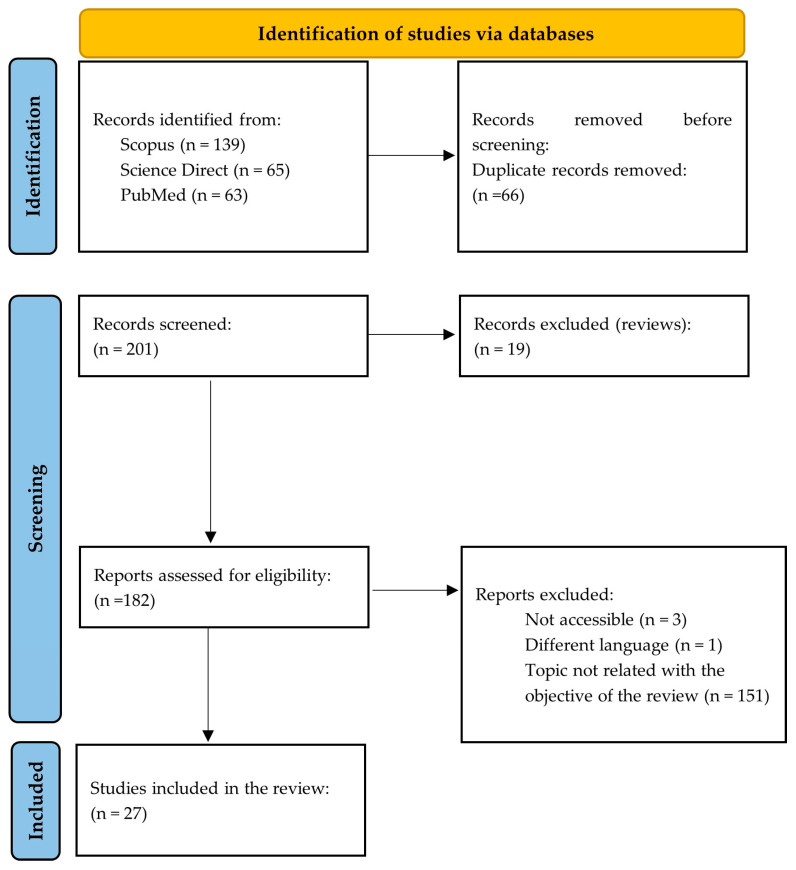
Scheme of the search strategy carried out for the systematic review according to the PRISMA Statement (adapted from [21]).

The search strategy is schematically described in Figure 1. 

## 3. Results

### 3.1. DPE 3D Printers

**Figure 2:**
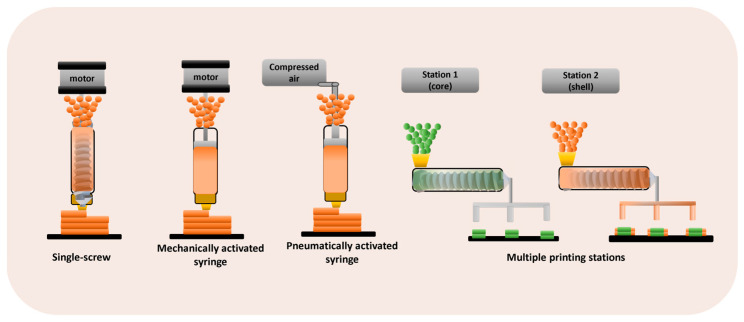
3D printers based on DPE technology.

Typically, DPE 3D printers are equipped with one or more printheads, each one incorporating a material extrusion procedure (single-screw extruder or a pneumatically or mechanically activated syringe) and a heated nozzle (Figure 2). 

**Figure 3:**
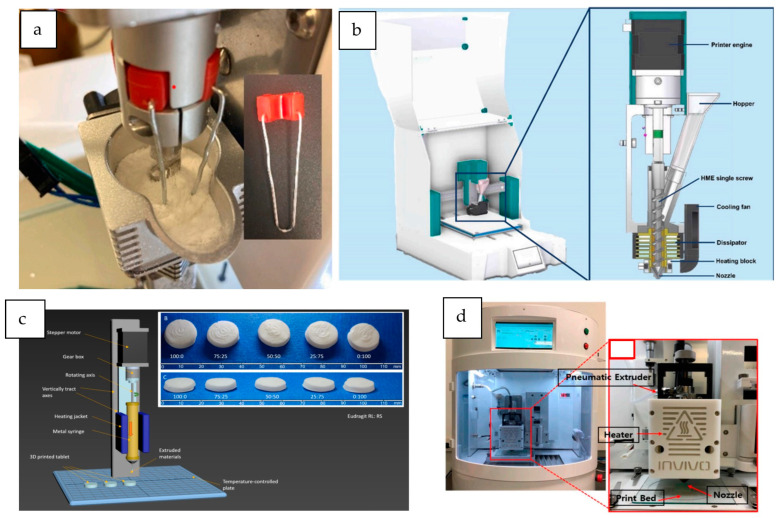
3D printers based on DPE technology. (**a**) M3DIMAKER DPE printhead, modified with a force feeder (from Rosch et al. [23], with permission); (**b**) extruder description of the 3DForMe printer, specifically designed for direct powder extrusion (from Pistone et al. [15], with permission); (**c**) schematic representation of the syringe-based mechanically activated Hyrel System 30 M equipped with a VOL-25 (Volcano) modular head (from Kuźmińska et al. [24], with permission); (**d**) ROKIT IN VIVO hot-melt air-extrusion 3D printer (from Cho et al. [25], with permission).

3D printers that are based on a single-screw extruder also incorporate a hopper. Multiple print heads are particularly useful for printing more complex geometries or multi-compartment structures [8,22]. The extrusion is controlled by the 3D printer software, and the extruder nozzle moves in the *X*, *Y*, and *Z* axes to construct objects in a layer-by-layer way [20]. Figure 3 shows examples of the different 3D printers used for DPE.

#### 3.1.1. Single-Screw DPE 3D Printers

The first DPE 3D printer specifically designed for the manufacture of pharmaceutical formulations was the M3DIMAKER, developed by FabRx (London, UK). This printer is the world’s first GMP-compliant pharmaceutical 3D printer with built-in quality control features for human precision medicine. It was equipped with a hopper and three interchangeable printheads. Specifically, a direct single-screw powder extruder with a nozzle aperture of 0.8 mm was used by Goyanes et al. [20] to directly print tablets. The design of the 3D printer consists of a single-screw extruder in which the rotation speed is controlled by the printer software. This 3D printer has also been used by other authors to successfully print tablets from a powder mixture of drugs, polymers, and plasticizers [23,26] or simply to obtain strands of the extruded material [27]. Rosch et al. [23] modified this printer with a custom-made stirrer (Figure 3a) to enhance the powder flow of the mixture.

Pediatric tablets of praziquantel in the form of amorphous solid dispersions (ASDs) were also prepared using the M3DIMAKER [28]. In this work, the physical powder mixture could not be processed directly due to the poor flow of the mixture and electrostatic forces. Therefore, processed material in the form of pellets obtained from HME extrudates and powders obtained from the milled pellets was used as feeding materials. Seoane-Viaño et al. [29] also had to perform a prior step, in particular a dry granulation step, to obtain a material with sufficient flowability for the successful direct extrusion and printing of tablets using this 3D printer. This was also the case of Pflieger et al. [30], who used granules previously prepared by HME as feeding material in the single-screw extrusion-based printer Flexdose^TM^ (Digital Health Systems GmbH (Schwäbisch Gmünd, Germany)) to produce immediate-release tablets. 

A similar design is shared by the 3D printer 3DForMe (Farmalabor, Canosa, Italy), which consists of a feed hopper and a single-screw extruder with a nozzle diameter of 0.8 or 0.4 mm (see Figure 3b). The extruder design is analogous to a single-screw HME, with the rotation speed controlled by the 3D printer’s software and the nozzle moving in three dimensions to allow layer-by-layer construction. This 3D printer has been used to produce drug delivery systems directly from powder blends [14,15,31].

The 3D Cultures (Philadelphia, PA, USA) have also developed a single-screw DPE 3D printer consisting of a hopper, a single-screw extruder, and a 1 mm nozzle. This 3D printer was used to develop sustained-release tablets with square geometries and transdermal patches [32,33].

Some authors have modified commercial FDM 3D printers to directly print pharmaceutical formulations in powder or pellet form. For instance, a da Vinci 1.0 FFF 3D printer (XYZPrinting, Ridderkerk, the Netherlands) was modified to incorporate a mini screw extrusion head (MAHOR.XYZ, Navarra, Spain) instead of the original extrusion head. The hopper was also modified to feed powder and pellets, including an agitator arm to improve the flowability of the powder blend towards the extruder [34].

A similar modification was made to an original FDM Prusa i3 MK2S 3D printer (Prusa Research, Prague, Czech Republic) to convert it into a powder-based 3D printer [35]. The modification consisted of adding a stainless steel hopper and a vertical single-screw enclosed in a barrel and heated by a band heater. A fan is positioned in front of the barrel and the hopper to control the temperature profile along the extruder. This prevents the powder mixture from melting prematurely and allows it to flow by gravity along the screw until it is melted and extruded through the nozzle.

Other authors designed a single-screw hot-end to be coupled to an in-house-developed FDM 3D printer [12,36]. This hot-end consists of a feeding section, a two-part barrel, a nozzle mounting, a printer nozzle, a single-screw extruder, and a PT 100 sensor to measure the extrusion temperature. These authors demonstrated the ability of this system to directly print several materials in powder form that are either not suitable for FDM or are difficult to process in their filament form. This increases the variety of pharmaceutical excipients that can be used in extrusion powder-based 3D printing.

A particular case is the 3D printer based on the melt extrusion deposition (MED^TM^) technique developed by Triastek (Nanjing, China). This is an extrusion-based GMP-compliant 3D printer that has been used to produce multi-component tablets with a variety of drug release profiles [22]. This equipment allows the continuous feeding of powdered material directly without the need for additional processing. It consists of a twin-screw feeding and mixing unit with different temperature zones where the material is mixed and melted, a material conveying module, several printing stations containing either a single or multiple nozzles through which the molten material is extruded, and an XYZ moving platform where the material is deposited in a layer-by-layer form. Each printing station is coordinated with each other to precisely build the different parts of a multi-component tablet with a predetermined design.

Moreover, this printer can be used to obtain a highly efficient production line with automated processes for continuous manufacturing, as it allows the integration of Process Analytical Technology (PAT) and Supervisory Control and Data Acquisition (SCADA) modules.

#### 3.1.2. 3D Printers Based on a Mechanical Syringe System

These systems apply a mechanical force directly to the syringe via a piston. Among the 3D printers based on a mechanically activated syringe extruder used to directly print powder formulations, two equipments manufactured by Hyrel 3D (Norcross, GA, USA) can be highlighted. Specifically, the type Hyrel 3D SR is a versatile unenclosed 3D printer that can be used in combination with different printheads that allow the 3D printing of a wide variety of materials. It has been recently used in combination with a TAM 15 extruder to produce spherical minitablets from a powder mixture [16,18]. This extruder consists of a 15 cc reservoir/plunger system with a 1 mm nozzle, in which the pressure is generated by a mechanical system based on a ball screw. It allows working up to about 200 °C.

Another type of Hyrel 3D printer is the System 30 M, which has been used to develop tablets and long-acting implants [24,37]. This other model of 3D printer is enclosed and also allows the combination of different printheads. In this case, a specific VOL-25 extruder was used, consisting of a 25 cc anodized aluminum syringe surrounded by a temperature-controlled heating jacket (Figure 3c). The semimolten material is extruded by a piston pushed by a computer-controlled stepper motor. The bottom of the syringe can hold a nozzle with a diameter of 1.3 mm [37] or a stainless steel luer-lock needle [24]. This syringe can be heated up to 100–110 °C. 

The extrusion in the MAM–II printer (Fuqifan Technology C., Ltd., Shangai, China) also takes place via a mechanically activated piston. This machine consists of a syringe heated by a thermo-couple with a 0.4 mm nozzle. It has been used to prepare sustained-release tablets [38].

#### 3.1.3. 3D Printers Based on a Pneumatic Syringe System

3D printers based on a syringe extruder connected to pressurized air directly from an air pressure source can also be a suitable strategy for the direct printing of powder formulations. In the reviewed studies, these 3D printers work with a wide range of air pressures (50–600 KPa), depending on the formulation requirements. Three printers can be classified in this group. One of them is the dual-nozzle pneumatic 3D bio-printer (BIO X and X2, Cellink, Gothenburg, Sweden), which was used to print controlled-release tablets with a core–shell structure in a single process [8]. This is a versatile bioprinter equipped with intelligent printhead mounts. In this study, an extruder consisting of stainless steel cylindrical cartridges screwed to the printhead nozzles (0.4 mm diameter) was used. The cartridges were connected to an external air compressor that provides high pressure (output limit of 700 kPa) and heated from the polymer’s glass transition temperature (T_g_) until the extrusion of the semisolid material under pressure. This bioprinter has also been used to produce raloxifene hydrochloride-loaded implants [19]. Other 3D printers based on this mechanism are the RegenHU Bioprinter (RegenHU, Villaz-St-Pierre, Switzerland) and the ROKIT INVIVO (ROKIT Healthcare, Seoul, Republic of Korea) (Figure 3d), both used in the preparation of different drug delivery systems [25,39,40,41]. Table 1 summarizes the main characteristics of the 3D printers used in the literature reviewed.

### 3.2. Critical Formulation and Printing Parameters 

One of the main advantages of DPE is that the mechanical properties of the raw material are not a limiting factor for its printability, as the rotation of the screw drives the movement of the material through the extruder [34]. Nevertheless, there are several critical parameters that must be considered to ensure a successful 3D printing process. 

#### 3.2.1. Amount of Feeding Material 

The DPE printing technique is suitable for small-scale batches. Single-screw 3D printers use between 3.5 and 10 g of feeding material [20,26]. An exception is the Hyrel 3D SR printer coupled with a TAM 15 extruder (Norcross, GA, USA) and based in a mechanically activated syringe, which allows the extrusion of 20 g batches [16,18]. Pneumatic printheads are used with even lower quantities of the starting material (maximum 5 g) [25,39,40]. Indeed, Wang et al. [8] found that the optimal load for the pneumatic printhead of the 3D bio-printer BIO X2 (Cellink, Gothenburg, Sweden) was around 2 g of powder per print. An excess of feeding powder resulted in a considerable heating time to ensure the melting of the material. Depending on the material, the heating time varied from 30 min to several hours [8]. This fact is advantageous when a small amount of drug is available but limits the number of dosage forms that can be printed per printing cycle.

#### 3.2.2. Rheological Parameters

Melt viscosity influences the extrusion temperature and the torque required to rotate the screw or the external force applied by the piston. An excessively high viscosity prevents the extrusion of the polymer through the hot-end, while a too-low viscosity of the melted polymer makes it impossible to obtain a solid structure after extrusion. 

Pflieger et al. [30] investigated the relationship between the viscosity of a material and its printability. For this purpose, they printed cylindrical tablets containing 25% *w*/*w* of metoprolol succinate and either Kollidon^®^ VA64 (KVA64) plasticized with polyethylenglycol (PEG) 6000 or Eudragit^®^ EPO and performed rheological measurements of the extruded blends using Small Amplitude Oscillatory Shear tests (SAOS). They identified two undesirable viscosity-related effects that hindered the continuous extrusion of the material: “clogging” and “polymer melt backflow”. These phenomena can be prevented by controlling the melt rheology of the material to be extruded. They found that a viscosity between 20 and 100 Pa·s would be appropriate for 3D printing and also demonstrated that the printing temperature, closely related to the viscosity of a material, is a key parameter that must be carefully studied. 

An extrusion multiplier, which is a tool that controls the volume of the extruded material per unit distance travelled by the printing head [42], can be used to modulate the flow of the material in order to reduce the variations in the melt viscosity of the different formulations and to achieve a continuous melt flow [34]. 

In the case of pneumatic DPE printers, they can only extrude semi-solid materials evenly and slowly, so viscosity is a critical factor (low-viscosity polymers are required). In most cases, the molten material is a non-Newtonian fluid, and its viscosity can increase or decrease under pressure. On the other hand, the use of plasticizers and lubricants or an increase in the drug loading, which in some cases reduces the viscosity of the mixture due to the plasticizing effect, decreases the temperature and pressure of the printing process [8,24,27].

The rheological behavior of the extruded material showed an important influence on its printability, particularly in terms of layer adhesion. Printlets prepared with EVA, a semi-crystalline copolymer of ethylene (E) and vinyl acetate (VA) containing a low percentage of VA, showed elastic behavior, resulting in poor adhesion of the first layer and weak bonding between layers. Conversely, the viscous behavior shown by formulations with a high percentage of VA resulted in adequate layer adhesion [34]. 

#### 3.2.3. Flowability

Flowability has been reported to be a challenge in DPE. A good flowability of the formulation is essential for an effective DPE process when using single-screw 3D printers, since the powder or pellets must flow smoothly through the screw to the heated nozzle in order to achieve continuous printing and good uniformity of mass and drug content in the dosage form. 

Goyanes et al. [20] observed a certain degree of variability in the mass of the 3D-printed tablets prepared (ranging from 309 mg to 348 mg), probably due to the different flow properties of the different hydroxypropylcellulose (HPC) grades employed. This was reflected in the deposition of different amounts of the blend by the extruder. 

In other cases, auxiliary excipients are also responsible for the poor flow. Racaniello et al. [31] found that the presence of cyclodextrin in the powder mixture increased the residence time in the printer due to powder wetting. However, the presence of the plasticizer and glidant polyethylene oxide (PEO) promoted the flow of the powder within the extruder. For this reason, formulations often include additives to improve the powder flow [23,28]. 

The importance of ensuring homogeneity of the powder particle size, which conditions a smooth powder flow, was highlighted by Pistone et al. [14,15], who repeated the sieving of each component of the mixture three times.

In order to improve the flowability of the formulations, some studies have compared the use of powders and processed materials to feeding. In this sense, the flow of different formulations in the form of physical mixtures, pellets obtained by HME, and milled pellets was analyzed [28]. In this study, only milled pellets provided a continuous flow through the screw. Moreover, flowability was also influenced by the drug load (mixtures containing 35% *w*/*w* praziquantel have a more reproducible flow than those with 50% *w*/*w* drug). A previous dry granulation step was also necessary to obtain a formulation based on Kollidon^®^ VA64 and mannitol with sufficient flowability to be printed by DPE [29].

It should be noted that the vertical orientation of the extruder also facilitates powder flow [14,20]. Nevertheless, some authors include an agitator arm or a stirring device to provide a constant powder feed rate. Hence, continuous and homogeneous transport of the powder blend through the extruder to the nozzle can be obtained, and tablets with uniform weight and drug content can be printed [23].

In another study, an agitator arm was also necessary to process a powder formulation containing 50% *w*/*w* of metoprolol tartrate and 50% *w*/*w* of EVA with an 18% content of VA. However, this agitator arm was not necessary when the same formulation was processed in pellet form [34]. 

#### 3.2.4. Printing and Platform Temperatures

Another important factor to control is the printing temperature. This temperature is determined by the melt viscosity of the polymer used, always bearing in mind that the drug and excipients used must be thermally stable at the processing temperature [8,23]. For this reason, it is essential to carry out studies on the thermal behaviour of the formulation components, usually using Differential Scanning Calorimetry (DSC) and Thermogravimetric Analysis (TGA). 

Different polymers that are either not suitable for FDM or show difficulties when used in filament form were tested by Feuerbach and Thommes [12] to demonstrate the ability of their homemade hot-end FDM printer to process these materials in powder form. These authors determined the minimum extrusion temperatures for these polymers and demonstrated that, for the crystalline and semi-crystalline polymers tested, the extrusion temperature had to be close to the melting point of the polymer, since at this point, the viscosity diminished significantly. For the amorphous polymers, the minimum extrusion temperature was between 50 and 100 °C above their glass transition temperature. Nevertheless, higher temperatures were used for the crystalline polymers EVA 4030AC and EVA1821A, which had to be printed at 30 °C and 80 °C above their melting points, respectively, to lower the viscosity and facilitate the extrusion process [32].

The porosity of the mixture has been identified as another factor affecting the printing temperature. The higher the porosity of the powder, the greater the increase in the contact surface area, which can lead to a reduction in sliding. As a result, slightly higher printing and platform temperatures are required to compensate for the poorer flowability [15].

The printing temperature is also strongly influenced by the incorporation of auxiliary excipients such as lubricants and plasticizers in the formulation [24]. For instance, Aguilar-de-Leyva et al. [27] demonstrated that adding 5% *w*/*w* of magnesium stearate to binary mixtures of polyurethane and theophylline reduced the DPE printing temperature from 150 °C–160 °C to 135 °C. 

The selection of the printing temperature can also be influenced by the melting temperature of the drug. Manini et al. [37] highlighted the importance of working above the melting point of the drug to prevent blockage of the materials at the nozzle, which could stop the process if the drug is not completely melted.

With respect to the platform temperature, in the majority of the reviewed studies, the platform is heated. It has been observed that the heated bed increases the adhesion between the platform and the structure that is being printed [32,40]. The range of temperatures used in the different studies is wide, varying from 25 °C to 80 °C. Although these temperatures are quite a bit lower than those utilized to heat the nozzle, the materials are exposed to them for a much longer period of time [43]. Therefore, the selection of this parameter must be carefully analyzed to avoid drug degradation.

#### 3.2.5. Other Printing Parameters

Other printing parameters, such as printing and travel speed, are critical factors for successful 3D printing by DPE. These parameters influence the mean residence time of the material subjected to high temperatures within the printer [43]. Hence, they can also influence drug degradation. Furthermore, the printing speed also affects the thickness of the strand and the resolution of the printed object [41]. The printing speed range used in the reviewed studies is quite wide (0.5 to 25 mm/s). Nevertheless, in the majority of the studies (17 of 25), a printing speed between 10 and 20 mm/s was utilized. With respect to the travel speed, a wide range of values (5–90 mm/s) was also used. However, these data are not always provided by the studies. 

In relation to the nozzle aperture, its value is closely related to the resolution of the produced object. This way, the smaller the nozzle diameter, the higher the resolution of the system [10]. Nozzle diameters between 0.2 and 1.3 mm are used in the reviewed studies. Therefore, bigger nozzle diameters are used in comparison with FDM [43].

In the case of printers based on a pneumatically activated syringe, printing pressure is another key parameter. In this type of machine, the flow of material initiates when determined pressure and temperature values are achieved. In addition, the higher the pressure applied, the higher the extrusion rate of the material. In the reviewed studies, the pressure applied to the formulations varied from 50 to 600 kPa. These values are similar to those used for SSE [44,45].

### 3.3. Drug Delivery Systems Based in DPE

**Figure 4:**
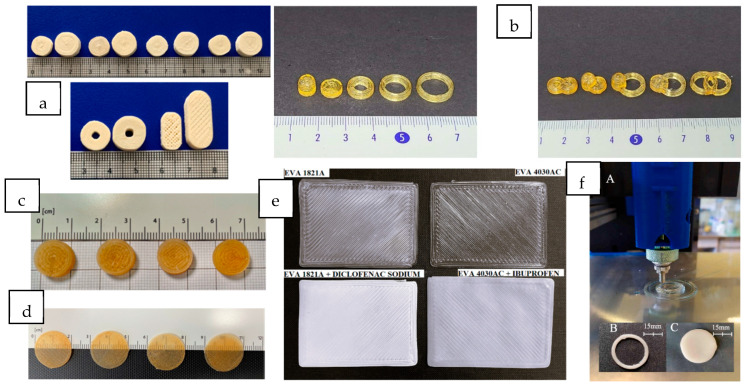
Drug delivery systems prepared by DPE. (**a**) Immediate-release printed tablets with different shapes: small and large cylinders, torus, and oblongs (scale in cm) (from Rosch et al. [23], with permission). (**b**) Sustained-release tablets with different shapes. From left to right, floating device, cylinder and rings, and combined geometries: cylinder–cylinder, cylinder-floating device, ring-floating device, ring–cylinder, and ring–ring (from Lee et al. [41], with permission). (**c**) Sustained-release cylindrical tablets based on different niclosamide formulations (from Pistone et al. [14], with permission). (**d**) Orodispersible films (ODFs) containing clobetasol (scale in cm) (from Racaniello et al. [31], with permission). (**e**) Top view of drug-loaded (on the bottom) and blank (on the top) EVA-based transdermal patches (from Maurizii et al. [32], with permission). (**f**) 3D printing process of a ring and disc-shaped implant on the Hyrel System 30M (from Manini et al. [37], with permission).

Due to its versatility, the DPE technique allows for the manufacture of a wide variety of dosage forms, covering the requirements of different pathologies. These systems can contain a great variety of drugs with a high drug load, up to 60% *w*/*w*. The following section describes the current 3D-printed drug delivery systems obtained by DPE, classified by their route of administration: oral, buccal, topical, and parenteral. Figure 4 shows some of these 3D-printed dosage forms.

#### 3.3.1. Oral Administration

The most commonly used geometrical shape for the manufacturing of oral dosage forms by DPE is a simple cylinder. Nevertheless, this 3D printing technology can lead to complex internal designs, resulting in structures such as core–shell and multi-compartment [8,14,15,16,20,22,23,24,26,28,29,30,34,35]. These modifications allow the combination of different drugs in the same dosage form and/or modulation of drug release profiles, as described in the following section.

- 3D-printed tablets

*Immediate drug release tablets*

Immediate drug release dosage forms can be successfully prepared by the DPE technique. The heating of components and their rapid solidification often entail a change from crystalline to amorphous forms of API, as confirmed by X-ray diffractograms and DSC results. This change leads to a higher solubility and dissolution rate of the drug.

One of the most commonly used polymers for this technique is Kollidon^®^ VA64, a water-soluble copolymer of N-vinylpyrrolidone and vinyl acetate (monomer ratio 6:4), also known as copovidone. Its low T_g_ (100 °C) makes it suitable for DPE, although it often requires the addition of a plasticizer. Recently, this polymer has been used to prepare immediate-release cylindrical tablets containing efavirenz (5–35% *w*/*w*) for the treatment of HIV [29]. The efavirenz granulates were formulated with Kollidon^®^ VA64 (40% *w*/*w*) and mannitol (20–55% *w*/*w*) and magnesium stearate (5% *w*/*w*) as plasticizers and were used as the feeding material for DPE. The printed systems released more than 80% of drug loads within the first 60 min. 

Copovidone has also been used in combination with the copolymer poly vinylalcohol-polyethylene glycol (Kollicoat^®^ IR) to prepare immediate-release 3D-printed tablets containing levodopa (28% *w*/*w*) and carbidopa (7.56% *w*/*w*) in two doses (60/15 mg and 170/42.5 mg). This combination is of clinical interest in the treatment of Parkinson’s disease [23]. Kollicoat^®^ IR is less brittle than copovidone, and its higher melt viscosity promotes adhesion on the built plate. It also improves the mechanical properties of the printed tablets. The mixture also requires the addition of mannitol (10% *w*/*w*) to provide the most reliable printing process and fast tablet disintegration. In this work, the influence of the tablet shape (cylinder, torus, and oblong) (Figure 4a) on the disintegration and dissolution behavior was investigated. The results showed faster dissolution for the oblong tablets due to their reduced infill. 

Kollidon^®^ VA64 has also been used in the manufacture by DPE of pediatric cylindrical tablets containing 35 and 50% *w*/*w* of praziquantel (100 and 150 mg, respectively) [28]. Surfactants (Span™ 20 or Kolliphor^®^ SLS Fine) were added to the formulations to increase the dissolution of the solid dispersions. Praziquantel is a Class II BCS drug characterized by a low drug solubility (0.02 mg/mL), an unpleasant taste, and variable dosing, great challenges that need to be overcome in the development of a pediatric oral formulation. Tablets were successfully produced from both pellets and powders obtained from HME extrudates. The results indicate that Praziquantel remained amorphous due to the HME treatment before printing, even after 3 months of storage at 25 °C/60% RH. Due to the formation of amorphous solid dispersions, in vitro praziquantel release was clearly improved. Moreover, the formulation of 3D-printed tablets was helpful for taste masking when compared to powder dosage forms. 

On the other hand, Kollidon^®^ VA64 has been used in combination with a hydrophilic matrix, such as HPC SSL, allowing milder processing conditions, such as lower temperatures and pressures, compared to the use of HPC as a single excipient. Fanous et al. [39] prepared oval tablets with a honeycomb infill pattern containing 10% *w*/*w* caffeine as a model drug. Different proportions of HPC (45–90% *w*/*w*), with or without Kollidon^®^ VA64 (31.5% *w*/*w*), and PEG 4000 (13.5% *w*/*w*) were used. Kollidon^®^ VA64 acts as a rapidly dissolving polymer, facilitating the dissolution process. The research demonstrates the effect of infill density and thus geometry on the release rate, as an increase in surface area results in greater water access and faster dissolution.

Formulations with 65% *w*/*w* of Kollidon^®^ VA64 and 5% *w*/*w* of PEG 1500 were used to obtain cylindrical tablets containing 25% *w*/*w* of metoprolol succinate. Complete drug release was achieved after 60 min [30]. In this work, the acrylic polymer Eudragit^®^ EPO was also tested, confirming its ability to produce immediate-release dosage forms by DPE. 

Among the acrylate derivatives, Eudragit^®^ E100 has also been evaluated by DPE to obtain 3D-printed cylindrical tablets. Jennotte et al. [35] used Eudragit E100 and Soluplus^®^ (SOL) (polyvinyl caprolactam–polyvinyl acetate–polyethylene glycol graft copolymer) in an 18–40% *w*/*w* range, due to their ability to increase the solubility of poorly soluble drugs, for the preparation of ASDs containing 10% *w*/*w* of cannabidiol (65 mg). These two polymers are not printable on their own because of their brittleness, so Polyox^®^ N10 (PEO) was added as a plasticizer (50–72% *w*/*w*, depending on the formulation). Cannabidiol was chosen as a BCS II model drug, and dissolution results showed an immediate drug release (80% of the drug released in 45 min) from the printed systems studied.

Some studies compare the behavior of different polymers processed by DPE in order to increase the dissolution rate of a poorly soluble drug in comparison with tablets obtained by direct compression. For instance, Kim et al. [40] prepared four formulations with dutasteride (1% *w*/*w*), Lutrol^®^ F 68 (10% *w*/*w*), and one of the following polymers (89% *w*/*w*): Soluplus^®^, Kollidon^®^ VA64, Eudragit^®^ EPO, and HPC. The authors managed to enhance the dissolution of the drug, which was in an amorphous state with Kollidon^®^ VA64, reaching above 80% of drugs released within 15 min. HPC also increased the dissolution rate when compared with directly compressed tablets. No significant differences were found for the other polymers. The authors also investigated four different 3D-geometries (cube, pyramid, hemisphere, and tube) to evaluate the effect of the surface area/volume (SA/V) ratio on the drug release profiles. It was demonstrated that the larger the SA/V ratio, the faster the drug release.

*Sustained drug release tablets*

Prolonged drug release from 3D-printed tablets can be achieved through different strategies. One of the most used is matrix formation using hydrophilic or hydrophobic polymers such as HPC, hydroxypropymethyl cellulose (HPMC), copovidone, EVA, methacrylate derivatives, etc. 

Among the hydrophilic polymers, HPC has been widely used in FDM 3D printing due to its suitable mechanical properties and ease of extrusion. Four different low-viscosity grades of HPC have been evaluated as matrix-forming excipients for DPE: HPC-UL (MW 20,000), HPC SSL (MW 40,000), HPC-SL (MW 100,000), and HPC-L (MW 140,000) [20,26]. These soluble polymers were used at a concentration of 65% *w*/*w* without any other adjuvant to prepare cylindrical tablets of itraconazole [20]. All the polymer grades were successfully printed, showing good adhesion between the printed layers. In addition, powder X-ray diffraction (PXRD) and DSC results suggested that the drug was amorphous in the formulations. In fact, the dissolution studies revealed a drug concentration 20 times higher than the itraconazole solubility. All printed formulations showed sustained release over more than 24 h, with a zero-order release profile during the first 8 h. It was also observed that the lower the molecular weight of the HPC, the faster the drug release. 

HPC-SL and HPC-L in 50–60% *w*/*w* were also used to prepare cylindrical tablets containing 5% *w*/*w* of tramadol hydrochloride with alcohol-resistant and abuse-deterrent properties. Formulations with 50% *w*/*w* of HPC also contained 40% *w*/*w* D-mannitol and 5% *w*/*w* magnesium stearate, while formulations with 60% *w*/*w* of HPC contained 20% *w*/*w* PEO, 10% *w*/*w* D-mannitol, and 5% *w*/*w* magnesium stearate [26]. A prolonged-release profile over 12 h was observed due to the formation of a thick viscous gel layer. The X-ray diffractograms of the tablets revealed no crystalline peaks corresponding to tramadol, suggesting that the drug was in an amorphous state. All the formulations exhibited alcohol-resistant and moderate abuse-deterrent properties, meeting the objectives of the study. This technique was advantageous when compared to other methods of production of this type of formulation, such as injection molding, which implies high costs in the production of reduced batches. 

Another hydrophilic polymer employed in DPE is polyvinylpyrrolidone (PVPK12). Lee et al. [41] used this polymer (75% *w*/*w*) with PEG 1500 (5% *w*/*w*) to prepare customizable modified-release solid dosage forms of ibuprofen (20% *w*/*w*) as a model API. Different shapes, such as cylinders, rings, and a floating dosage form with a cavity, and the fusion of two different geometries in the same dosage form were evaluated (Figure 4b). Diffractogram results showed that ibuprofen was molecularly dispersed in the extrudates in an amorphous form.

HPMC HME 15 LV is used as a water-soluble cellulosic polymer to enhance the solubility of poorly soluble drugs by maintaining stable solid dispersions and inhibiting API crystallization. Moreover, it has a polymer substitution structural design that facilitates thermal processability by HME, making this excipient an excellent candidate to produce stable ASDs with poorly soluble APIs. Thus, formulations containing 10% *w*/*w* of niclosamide (50 mg) and HPMC HME 15 LV (40–90% *w*/*w*) were processed by DPE to obtain cylindrical tablets (see Figure 4c) [14]. The effect of the addition of 4.5% *w*/*w* of PEG 6000 to the previous formulations was also studied. The resulting systems showed a prolonged and complete drug release over 48 h, following zero-order release kinetics during the first 8 h. The presence of hydroxypropyl-β-cyclodextrin (HP-β-CD) (47% *w*/*w*) in the mixture was also investigated to improve the poor water solubility of niclosamide. The results show that the drug release from the dosage forms occurred through the matrix swelling and solubilization, since the combination of HPMC and HP-β-CD showed a synergistic effect improving the aqueous solubility of the drug. Furthermore, DSC, Fourier transform infrared spectroscopy (FT-IR) and PXRD results confirmed the amorphization of the drug during the printing process, which also contributed to the increase in drug solubility. The amorphous state of the drug is maintained after 3 months of storage at 25 °C and 60% RH [14].

Copovidone has also shown the ability to produce sustained-release tablets by DPE. Liu et al. [38] used a mixture of copovidone (56% *w*/*w*), acetaminophen (35% *w*/*w*), and the plasticizer Poloxamer^®^ 407 (9% *w*/*w*) to prepare sustained-release cylindrical tablets of three different commercial strengths (650 mg, 500 mg, and 350 mg).

On the other hand, hydrophobic polymers such as EVA are also used as matrix polymers. Almeida et al. [46] used EVA without any other adjuvant excipient to produce sustained-release dosage forms via HME. This fact, along with the flexible physicochemical properties of this polymer, makes it a potential candidate to be employed with hot-processing techniques such as DPE. The physical and mechanical properties of the copolymer differ with the VA content, being rigid at low VA contents and rubbery at high VA contents. The 3D-printed cylindrical tablets containing metoprolol tartrate (50% *w*/*w*) and EVA with different VA content (50% *w*/*w*) were manufactured by DPE [34]. All pellet formulations were successfully printed, unlike filaments, which could only be printed by FDM in the case of EVA with 18% VA content due to mechanical and rheological problems. Feuerbach et al. [12] were also able to process formulations containing 100% of EVA (28% VA content) in powder form with a homemade hot-end adapted to a conventional FDM printer, despite its unsuitability in filament form [36]. Therefore, it seems that the mechanical and rheological properties of the raw materials are less limiting factors for DPE. All tablets showed high content uniformity and a homogeneous distribution of metoprolol, which remained crystalline in the EVA matrix. A prolonged drug release profile over 12–24 h was observed for all preparations. 

Poly(meth)acrylates, such as Eudragit^®^ RL and Eudragit^®^ RS, are other hydrophobic polymers that have also been used to produce sustained-release cylindrical tablets by DPE. Kuźmińska et al. [24] prepared different formulations containing theophylline (40% *w*/*w*) and varying the proportion of Eudragit^®^ RL and Eudragit^®^ RS (0–40% *w*/*w*). It was possible to modulate the theophylline release profile by changing the methacrylate polymer ratios. The release rate of theophylline decreased as the Eudragit^®^ RS content increased due to the lower hydrophilicity of this polymer. The formulations required an excess of the plasticizer triethyl citrate (TEC) (12%), chosen by its miscibility with the acrylate polymers, in combination with glycerol monostearate (8%), to be extruded. The use of these plasticizers and lubricants also allowed the blends to be printed at lower temperatures (up to 110 °C maximum). 

Finally, poly (3-hydroxybutyrate) (PHB) has also been investigated as a promising alternative biopolymer for DPE [33]. This thermoplastic aliphatic polyester is biodegradable, biocompatible, and environmentally friendly, with a high crystallinity (60–70% *w*/*w*) and a molecular weight > 400 kDa. The authors prepared three different model square-shaped systems with acetaminophen as the model drug (10, 20, and 30% *w*/*w*). Dissolution results showed a prolonged release for these systems, following a diffusion process.

- Core–shell 3D-printed tablets

Core–shell tablets are a common design in 3D printing technology, particularly in FDM [47,48,49]. This structure offers the possibility to modulate the drug release profile by only changing the characteristics of the drug-free shell, such as the thickness or composition, without modifying the size of the core containing the drug [50]. Recently, DPE has been used to fabricate core–shell geometry controlled-release tablets containing acetaminophen (40% *w*/*w*) in the core [8]. Acetaminophen was chosen as a model drug due to its compatibility and its ability to form hydrogen bonds with polymers, making it widely used in the production of solid dispersions. Moreover, it is thermally stable at temperatures below 260 °C. During printing at 140 °C, acetaminophen (T_m_ 172 °C) was converted from its crystalline to the amorphous state, being dissolved in molten Kollidon^®^ VA64. This polymer was used as the core filler (60% *w*/*w*) due to its water solubility and its application in the manufacture of ASDs. The drug dissolution profiles showed a complete drug release from the core tablet in 5 h. 

The immediate-, sustained-, and delayed-release polymers Eudragit^®^ E PO, Eudragit^®^ RS PO, and HPMCAS LG were used as shell polymers. Eudragit^®^ E PO is a copolymer of dimethylaminoethyl methacrylate and other neutral methacrylic acid esters that dissolves at pH < 5 and has great printability. Eudragit^®^ RS PO is a copolymer synthesized from acrylic acid and methacrylic acid esters, widely used for extended release due to its low permeability and water-insoluble character. HPMCAS LG is a mixture of acetic acid and monosuccinic acid esters of HPMC in granular form, often used as an enteric coating due to its pH-sensitive character (soluble at pH > 5.5) and rapid dissolution in the upper intestine. It is also useful in the formulation of ASDs. HPMCAS LG exhibits a high melt viscosity, being necessary a plasticizer for processing. Hence, PEG 4500 was added as a plasticizer agent (20% *w*/*w*) in formulations containing HPMCAS LG and as a pore-forming agent (10–20% *w*/*w*) in those based on Eudragit^®^ RS PO.

As expected, core–shell tablets with pure Eudragit^®^ E PO showed a lower drug release rate than the core tablet, but the dissolution of the shell and the core was not sequential, and water uptake into the inner core was facilitated by the interlayer spaces of the shell. The presence of PEG 4500 in the HPMCAS LG shell formulation increased its porosity, and these tablets exhibited an extended rather than delayed release. Coating with Eudragit^®^ RS PO and PEG 4500 was more effective to protect the core, showing these systems zero-order drug release kinetics over 20 h. The authors demonstrated that the dissolution rate could be modulated by varying the shell composition.

- 3D-printed minitablets

Minitablets are small tablets, typically with a diameter lower than 4 mm. The small size of the tablets offers several advantages, as they are easy to swallow and commonly accepted by children [51]. They also offer the possibility of being incorporated into a hard capsule, where different formulations can be combined. 

DPE 3D printing has been used to manufacture 3D-printed spherical minitablets containing 25% *w*/*w* of either ritonavir or lopinavir (40 mg) to develop a personalized solid dosage pediatric formulation for HIV treatment [18]. HPMCAS LG grade (51.75% *w*/*w*) was used as the matrix forming polymer. Other components of the formulation were the plasticizer PEG 4000 (22.5% *w*/*w*) and the lubricant magnesium stearate (0.75% *w*/*w*). Hydrogen bonding interactions between drugs, HPMCAS and PEG 4000, resulted in the production of ASDs with a sustained-release zero-order kinetics profile at pH 6.8. The fraction of the drugs dissolved remained in solution despite their low solubility in the intestinal pH. The minitablets showed high uniformity in drug content, weight, diameter, and density. 

The fabrication of these dosage forms was rapid, and the minitablets produced could be fitted in a size 0 hard capsule. They can be easily swallowed by pediatric patients, allowing a versatile administration of the drug adapted to their weight. An accurate dose can be achieved by changing the diameter of the minitablet or the number of minitablets administered. 

In another study, cylindrical minitablets loaded with budesonide and intended for the pediatric treatment of eosinophilic colitis were manufactured by DPE [15]. Three different formulations containing 0.59% *w*/*w* of budesonide (1 mg) were prepared. The results obtained demonstrated the amorphization of the drug during the printing process. HPMC HME 15 LV (Affinisol^®^), a cellulose derivative with thermal properties suitable for extrusion-based 3D printing processes, was used as the matrix polymer in a 41–75% *w*/*w*, depending on the formulation. All the mixtures included an adjuvant blend (8–20% *w*/*w*) consisting of sodium bicarbonate, citric acid, and tartaric acid to accelerate the HPMC dissolution and prevent the drug recrystallization in the gel layer. HP-β-CD (47% *w*/*w*) was added to improve the solubility of the active ingredient, and PEG 6000 (2.99–3.97% *w*/*w*) was used as a plasticizer. The time required to complete the printing process of each tablet was approximately 3 min, and the minitablets obtained showed good mechanical and physical properties, as well as a sustained-release profile. To achieve a colon-specific drug release, the minitablets were coated in a fluidized bed with Eudragit^®^ FS30D.

Cylindrical minitablets containing nifedipine for the treatment of hypertension were also prepared by DPE [16]. The drug loading of each printed system was 25% *w*/*w* (20 mg). Other components of the formulation were HPC LF (40% *w*/*w*) and HPMCAS LG (19% *w*/*w*) as rate-controlling polymers, PEG 4000 (15% *w*/*w*) as plasticizer, and magnesium stearate (1% *w*/*w*) as lubricant. The amorphization of the drug was observed after the printing process, and a prolonged drug release profile based on erosion was obtained over 24 h. This release profile was preferable to that exhibited by commercially available formulations that disintegrate quickly, showing a pronounced burst effect. The printing time of each tablet was 2 min, and the dose could be easily adjusted to the patient’s needs by simply modifying the tablet height, facilitating the production of personalized medicines. The minitablets obtained can be fitted inside a 0-size capsule with other minitablets containing other active ingredients, which would allow the preparation of a multidrug solid dosage form. A minimal risk of physicochemical interactions between drugs is expected, as each drug is contained in an independent tablet.

- Multicomponent tablets

Multicomponent tablets offer the possibility of incorporating different drugs with different release kinetics, even if they show incompatibility issues, independently in the same dosage form. Multimaterial printing also allows the design of different structures within the dosage form. In this sense, compartmental-model tablets with six different designs were prepared by melt extrusion deposition (MED^TM^) employing a GMP-compliant (MED^TM^) 3D printer with multiple printing stations [22]: multilayered drug compartment in core–shell structure tablets, modified release tablets in core–shell structure with a delay layer, modified release tablets in core–shell structure with a pH-responsive layer, multi-compartment tablet for independent release of drug combinations, multi-component tablet for concurrent release of immediate-release formulation and extended release formulation and single-compartment tablet for sequential release of immediate-release formulation and extended release formulation. Different model drugs (metoprolol succinate, tofacitinib citrate, levodopa and carbidopa, topiramate and clonidine hydrochloride) and a wide range of polymers (Eudragit^®^ RS PO, HPC EF and JF, HPMCAS HG and LG, ethyl cellulose (EC), and Kollidon^®^ VA64) and auxiliary excipients (PEG 400, PEG 1500, PEG 8000, stearic acid, TEC, and croscarmellose sodium) were employed. It was demonstrated that the direct 3D printing of compartmental designs allowed the modulation of the release onset time, release kinetics, and release duration with high precision and reproducibility. Furthermore, the authors of this study proposed a product development approach called 3D printing formulation by design (3DPFbD^®^) to offer a simple and effective tool for the rapid and efficient development of pharmaceutical dosage forms.

#### 3.3.2. Buccal Administration

- Orodispersible Films (ODFs)

Among the existing dosage forms for buccal applications, biocompatible and biodegradable mucoadhesive films are the most preferred systems due to their versatility, adaptability, physical flexibility, comfort, lightness, acceptability, ability to withstand mechanical stress, and adjustable size [52]. For those reasons, orodispersible mucoadhesive cylindrical films containing 0.20% *w*/*w* (125 µg/dose) of clobetasol propionate for the pediatric treatment of Oral Lichen Planus, a rare chronic disease, were prepared by DPE (Figure 4d) [31]. Clobetasol is a corticosteroid for topical administration with strong anti-inflammatory, antipyretic, and vasoconstrictive properties. However, its therapeutic efficacy is limited by its low aqueous solubility (2 µg/mL). PEO N10, with a molecular weight of about 100,000 Da (71.4 to 81.45% *w*/*w*, depending on the formulation), was selected for its mucoadhesive properties. It was blended with chitosan of low molecular weight (15–25% *w*/*w*, depending on the formulation), which was also selected for its mucoadhesiveness and biocompatibility. Affinisol^®^, which also exhibits mucoadhesive characteristics, was used (0.35% *w*/*w*) along with HP-β-CD (3% *w*/*w*) to increase the drug’s solubility. The homogeneous distribution of clobetasol inside the printed films was demonstrated by chemical microanalysis. During printing at 170 °C, the drug (T_m_ 220 °C) suffered a partial amorphization or complexation with HP-β-CD. The hydrophilic polymers (HPMC and PEO) and HP-β-CD showed a synergistic effect on improving clobetasol solubility. The presence of chitosan in the formulations enhanced the mucoadhesive properties of the films, as confirmed by permeation and retention studies through porcine mucosae. The obtained films showed an elastic and tenacious structure, sustained drug release over 20–30 min, and were stable for 3 months at 25 °C and 60% RH. 

The poorly water-soluble drug olanzapine, an atypical antipsychotic, was also formulated in orodispersible films [25]. The hydrophilic film-forming polymer for ODFs, PEO N-10 (70–75% *w*/*w*, depending on the formulation), was used due to its mechanically favorable flexibility. Kollidon^®^ VA64 (20% *w*/*w*) was also included in the formulation due to its ability to form ASDs by HME and to provide immediate release. Either Poloxamer^®^ 407 or Poloxamer^®^ 188 (5% *w*/*w*) were used as plasticizers due to their ability to increase the dissolution and disintegration rates of ODFs. Square-shaped films of 100 mg were obtained. DSC and PXRD results indicate the amorphization of olanzapine within the polymeric carriers after printing. All films showed fast disintegration times within 22 s and an immediate drug release.

#### 3.3.3. Transdermal Route

- Patches

Other routes of administration, such as the transdermal route, have also been targeted for the preparation of dosage forms by DPE. Transdermal patches based on EVA 4030AC and EVA 1821A loaded with ibuprofen and diclofenac sodium (30% *w*/*w* theoretical content), respectively, were developed by DPE (Figure 4e) [32]. Increasing the VA content decreases the flexibility, stress-crack resistance, toughness, and melting point of the polymer. Therefore, EVA 1821A (18% VA) has a higher melting temperature than EVA 4030AC (40% VA). These polymers are good candidates for 3D printing due to their low glass transition and easy extrudability without the addition of plasticizers.

Both formulations were successfully processed by DPE. The patches exhibited uniform weight and dimensions, indicating good reproducibility of the printing process. Although neither of the two final dosage forms achieved the theoretical 30% *w*/*w* loading, the amount of diclofenac sodium was lower than that of ibuprofen, which was attributed to the worse flowability of EVA 1821A during the 3D printing process. The patches produced with both formulations showed a good degree of flexibility and were suitable for use and transport. With regards to dissolution, an initial burst release was observed during the first 6 h, which was attributed to the dissolution of the drug on the surface of the patch. After that, a slower release was observed, which was attributed to the drug diffusion through the polymer matrix. The cumulative release of ibuprofen and diclofenac after 48 h was 74.5% and 12.6%, respectively. In our opinion, the low percentage of diclofenac released could be explained by the low drug loading achieved. This fact could lead to the existence of isolated clusters of drugs that do not connect with the exterior. Moreover, the low VA content of EVA 1821A reduces the microporosity of the matrix and therefore decreases the permeability of the polymer and the release rate. 

#### 3.3.4. Parenteral

- Implants

Implants are drug delivery systems consisting of a polymeric matrix or reservoir that allows targeted and prolonged drug delivery and are usually inserted intradermal or subcutaneously. These dosage forms produce therapeutic effects with lower drug concentrations, minimizing potential side-effects of therapy and offering the opportunity for increased patient compliance. This type of system is also employed to deliver drugs that cannot be administered orally [53,54].

Long-term implants containing 5 and 10% *w*/*w* of the poorly soluble drug paliperidone and polycaprolactone (PCL) as matrix-forming polymers were also prepared by DPE [37]. PCL is a semi-crystalline polyester with a melting point of around 61 °C, which allows for its processability at low temperatures. This fact, together with its excellent biocompatibility and slow in vivo degradation, makes it an excellent option for its use in prolonged-release drug delivery systems [55]. Before the 3D printing process, the blends were prepared by two methods: mortar and pestle and cryogenic milling. Paliperidone was amorphized during the cryogenic milling, and this state was maintained after printing. Two designs (ring and disc) were developed (see Figure 4f). All formulations prepared showed prolonged drug release for more than 3 months, and it was demonstrated that the drug release profile could be modulated as a function of the implant’s shape. Therefore, these dosage forms could be an interesting alternative to enhance the compliance of schizophrenic patients [37].

PCL (45% *w*/*w*) was also used for the manufacture of long-term subdermal implants containing raloxifene (10% *w*/*w*) and PEO N80 (45% *w*/*w*) by DPE. Three different shapes (cuboid, cylinders, and tubes) were printed, and a partial amorphization of the drug was confirmed by DSC and PXRD analysis. All 3D-printed structures showed a prolonged-release profile, with the cumulative percent released at the end of the 30-day study being 40.6% for the cylindrical-shaped implant, 35.6% for the tube-shaped implant, and 29.0% for the cuboid-shaped implant [19].

## 4. Discussion

Among the extrusion-based 3D printing techniques, DPE has broken recently in the production of pharmaceutical formulations with the aim of avoiding the limiting step of producing hot-melt extruded filaments, allowing the direct extrusion of a mixture in powder or pellet form. The existing DPE 3D printers are based on three different mechanisms, but according to the reviewed works, most of the studies analyzed (16 of 27) employ a single-screw 3D printer. The functioning of this machine is based on a single-screw HME coupled to a heated nozzle that moves in the three dimensions of the space. Therefore, it can be considered a fusion between the HME technique and the FDM technology. It has been observed that the flowability of the mixture is a key parameter when this type of printer is used. For this reason, sometimes it is not possible to use the formulation directly in powder form, but it is necessary to employ granules, pellets, or even powder obtained from milled pellets. These pellets or granules have been previously prepared from cut filaments obtained by HME [28,30,34] or by a previous dry granulation step [29]. In these cases, the technique is no longer a single-step process, losing one of the main advantages of the DPE. Nevertheless, the homogeneity of the filament diameter, which is a limiting factor in FDM [43,56,57,58], does not affect it in these circumstances. 

On the contrary, in all the studies that employ a 3D printer based on a mechanically or pneumatically activated syringe, the use of powder as feeding material is possible. In these cases, since the formulation melts directly in the cartridge, the vertical flow of the mixture is not as important as other factors such as the melt viscosity. 

In some cases, the studies that employ syringe-based printers use different terms to refer to the DPE technique. This way, expressions such as hot-melt pneumatic extrusion, hot-melt 3D extrusion, hot-melt extrusion 3D printing, pressure extrusion-based printing (PEBP), and melt extrusion deposition (MED^TM^) are utilized to refer to the direct extrusion of a powdered formulation at temperatures quite higher than those used in the SSE technique [22,25,37,38,40,41].

In relation to the materials employed in the different formulations, only in a few studies the direct extrusion of a binary mixture of drug and polymer (HPC, EVA, PCL, and PHB) is possible [19,20,32,33,37], but in most cases, the use of auxiliary excipients is needed (see Table 2).

Among these auxiliary excipients, plasticizers are the most widely employed in proportions ranging from 3% to 55% *w*/*w*. In particular, PEGs of different molecular weights are the most commonly used. The use of a plasticizer lowers the glass transition temperature of the polymer, enhancing its melt flow properties and allowing its processing at lower temperatures. This type of excipient is also frequently used in FDM for the same reason [59,60]. In a few studies, the inclusion of a lubricant is also necessary to promote the powder flow in the printer. Magnesium stearate is used in all cases in a proportion ranging between 0.75 and 5% *w*/*w*. This lubricant is also widely employed in HME-FDM for improving the extrusion of filaments, due to the enhanced flow of the powder mixture and the reduction of the frictional forces with the HME screw [61,62]. Dehydrating and glidant agents in a low proportion were only used in one case to counteract the moisture of the powder blend [14]. 

Other auxiliary excipients are employed to modulate the drug release properties of the dosage forms prepared. This is the case of the use of croscarmellose sodium as a disintegrant to obtain immediate-release compartments in the preparation of multicomponent tablets [22]. With the aim of accelerating the drug release rate, solubility enhancers such as HP-β-CD, Soluplus^®^, Kolliphor^®^ SLS fine, Span^TM^ 20, or Lutrol^®^ F68 are used for the formulation of BCS class II drugs such as niclosamide, budesonide, clobetasol, praziquantel, dutasteride, or cannabidiol [14,15,28,31,35,40]. In this sense, it should be noted that in most of the reviewed works, the API suffers an amorphization process during the extrusion and is present in the form of ASDs. There are also a few works in which the API suffers a partial amorphization [19,27,31]. This fact leads to an increase in the drug solubility and dissolution rate, which is beneficial in the case of the formulation of BCS class II drugs. Therefore, DPE can be employed as an alternative to the existing methods for the preparation of ASDs [40]. 

Although the number of works using DPE is limited, a high variety of drug delivery systems containing a wide array of different drugs intended for different administration routes have been produced (see Table 2). Due to the ease of use, convenience, safe, and patient compliance of oral administration, it continues to be the most explored route. Thus, the number of oral drug delivery systems manufactured by DPE is substantially greater than for other administration routes. While the oral dosage forms most frequently manufactured are immediate and sustained-release cylindrical tablets, various geometries such as cube, pyramid, square, oval, or ring have also been successfully printed [23,33,39,40,41]. The feasibility of producing other types of tablets, like minitablets [15,16,18], core–shell tablets [8], or multicomponent tablets [22], by DPE has also been demonstrated. High breaking force and low friability values for the oral 3D-printed systems prepared by DPE, comparable to those prepared by FDM, have been obtained [20,26].

In addition to oral administration, other routes have also been investigated using DPE, such as parenteral, buccal, and transdermal. Thus, ODFs and long-term implants of different shapes, as well as transdermal patches, have also been prepared by this technique [19,25,31,32,37]. 

The versatility of this technology as a tool to produce personalized drug delivery systems for the treatment of different diseases has been proven.

## 5. Conclusions

In this review article, the application of the 3D printing technology DPE to the manufacture of drug delivery systems has been analyzed. Since its first application in the pharmaceutical field in 2019, 27 articles based on this technology have been published. The operation of the different 3D printers used as well as the role of key parameters such as excipients, temperature, rheological properties, or flowability have been analyzed. The formulation, geometrical shape, and drug release kinetics of the drug delivery systems prepared have also been studied. DPE has demonstrated itself to be an effective technique for the manufacture of a wide range of dosage forms containing varied drugs with different release profiles intended for different routes of administration. The possibility of developing tailored medicines adapted to the drug requirements of patients affected by different pathologies has also been pointed out. The circumvention of many of the limitations of other extrusion-based 3D printing techniques, such as FDM and SSE, proposes DPE as an innovative, time, and cost-efficient one-step printing process.

## 6. Future Directions

In the last 5 years, DPE has burst onto the scene with force among 3D printing techniques applied to the development of drug delivery systems. Since then, an important number of studies dealing with the development of different dosage forms containing a wide variety of drugs and excipients have been carried out. The feasibility of this technology has been extensively demonstrated. The following are some of the aspects that must be considered before integrating this technique into clinical practice:

- Economic and Environmental Impact: 3D printing techniques have great potential in the development of sustainable drug delivery systems. Although 3D printing is not a waste-free manufacturing technique, it generates less waste than traditional manufacturing processes, decreasing its environmental footprint. Sustainability of DPE 3D printing could be achieved through optimized material usage and recycling. The use of biodegradable, biocompatible, and eco-friendly polymers could help minimize environmental impact. Besides its sustainability, single-step DPE appears to be an innovative 3D printing technique for small-scale manufacturing of personalized dosage forms compared with rigid and multi-step conventional manufacturing processes. However, to improve its cost-efficiency, it would be essential to develop 3D printers with higher printing speeds. With regards to productivity and time efficiency, DPE also has clear advantages compared with other 3D printing material-extrusion techniques, such as FDM. Since there is no need for filament feedstock preparation, formulation development will be accelerated, and the cost of production of drug delivery systems will be reduced. Moreover, the small amount of powder blend required (only a few grams) also contributes to the cost-efficiency of this manufacturing process. This fact is particularly advantageous when processing high-cost drugs, such as orphan drugs. Moreover, in contrast with other 3D printing techniques such as SSE, which usually need a post-processing drying step, drug delivery systems prepared by DPE have no post-printing requirements, shortening the time needed to prepare the medical prescription [16,17,23,24,33,63,64,65].
- Regulatory Challenges: The future of DPE will probably be the application in clinical settings such as hospitals and compounding pharmacies, rather than the mass production of medicines. In this context, regulatory challenges need to be addressed. Due to the flexible and on-demand production process, standard regulations could not be applied, so new regulatory guidelines should be implemented. Another key aspect is the standardization of DPE 3D printers to meet Good Manufacturing Practice (GMP) requirements, which is critical to guaranteeing the production of high-quality and safe medicines. In fact, some GMP-compliant DPE printers have been recently introduced. Moreover, the limited number of preclinical and clinical studies performed currently is another hurdle for the regulatory approval of 3D-printed pharmaceuticals [10,17,61,64,65].
- Long-Term Stability Issues: Since 3D-printed medicines are expected to be produced on-demand and dispensed extemporaneously, the long-term stability of the product should not be a significant problem. Nevertheless, several studies have carried out stability analyses, most of them conducted over a period of 3 months at 25 °C and 60% RH. The results of these studies highlight the absence of recrystallization and/or degradation of the drug [14,15,28,31].
- Challenges with Personalized Medicines: DPE offers unique benefits in the tailoring of the dosage forms. Pediatric and geriatric patients present physiological particularities that require dose adjustment. Furthermore, children have swallowing problems and are especially sensitive to the taste of the formulation. DPE has demonstrated its ability to solve these issues. Hospital pharmacy services have the necessary infrastructure to accommodate this technology. Due to their compact and user-friendly nature, DPE printers are particularly suitable to produce small batches of customized formulations in community and hospital pharmacy settings. The decentralized manufacture of pharmaceuticals could be beneficial from a logistical perspective, as transport costs and storage time could be reduced. However, it would be of outmost importance to protect patient privacy and guarantee data security in the implementation of DPE technology in decentralized healthcare locations [18,28,65,66,67,68].

## Figures and Tables

**Table 1:** Main characteristics of the DPE 3D printers used in the literature reviewed.

DPE 3D Printer	Extrusion Mechanism	Feeding Materials	References
M3DIMAKER (FabRx, London, UK)	Single-screw	Powder	[20,23,26,27]
		HME pellets	[28]
		Powder from milled pellets	
		Granules obtained by dry granulation process	[29]
Flexdose^TM^ printer (DiHeSys, Schwäbisch Gmünd, Germany)	Single-screw	HME granules	[30]
3DForMe (Farmalabor, Canosa, Italy)	Single-screw	Powder	[14,15,31]
Single-screw powder extruder (3D Cultures, Philadelphia, PA, USA)	Single-screw	Powder	[32,33]
Modified da Vinci 1.0 FFF 3D printer (XYZPrinting, Ridderkerk, The Netherlands)	Single-screw	Powder/pellets obtained by HME	[34]
Modified Prusa i3 MK2S 3D printer (Prusa Research, Prague, Czech Republic)	Single-screw	Powder	[35]
In-house developed FDM 3D printer	Single-screw	Powder	[12,36]
MED^TM^ 3D printer (Triastek, Nanjing, China)	Twin-screw	Powder	[22]
Hyrel 3D printer (Norcross, GA, USA) SR model coupled with a TAM 15 extruder	Mechanically activated syringe	Powder	[16,18]
30 M model coupled with a VOL-25 printhead			[24,37]
MAM—II printer (Fuqifan Technology C., Ltd., Shangai, China)	Mechanically activated syringe	Powder	[38]
3D bio-printer (BIO X and BIO X2) (Cellink, Gothenburg, Sweden)	Pneumatically activated syringe	Powder	[8,19]
RegenHU Bioprinter (RegenHU, Villaz-St-Pierre, Switzerland)	Pneumatically activated syringe	Powder	[39]
ROKIT INVIVO (ROKIT Healthcare, Seoul, Republic of Korea)	Pneumatically activated syringe	Powder	[25,40,41]

**Table 2:** Summary of the different drug delivery systems prepared by DPE.

Printing Technology	Geometrical Shape	Drug (% *w*/*w*)	Polymers (% *w*/*w*)	Auxiliar Excipients	Printing/Platform Temperatures	Reference
Immediate drug release 3D-printed tablets	Cylinder	Efavirenz (5–35%)	Kollidon^®^ VA64 (40%)	Mannitol 100 (20–55%)	180–190	[29]
				Magnesium stearate (5%)		
	Cylinder	Levodopa (28%), Carbidopa (7.56%)	Kollidon^®^ VA64 (20–30%)	Mannitol (10%)/	180	[23]
	Torus		Kollicoat^®^ IR (24.44–34.44%)	Compressol^®^ SM (10%)		
	Oblong					
	Cylinder	Praziquantel (35–50%)	Kollidon^®^ VA64 (50–60%)	Kolliphor^®^ SLS fine (5%)/	130–140	[28]
				Span^TM^ 20 (5%)		
	Oval	Caffeine (10%)	HPC SSL (45–90%)	PEG 4000 (13.5%)	155–180	[39]
			Kollidon^®^ VA64 (31.5%)			
	Cylinder	Metoprolol succinate (25%)	Kollidon^®^ VA64 (65%)	PEG 1500 (5%)	140/50	[30]
			Eudragit^®^ EPO (75%)		140/50	
	Cylinder	Cannabidiol (10%)	Eudragit^®^ E100 (18–40%)	PEO (49.5–72%)	155/25	[35]
			Soluplus^®^ (18–40%)			
	Cube	Dutasteride (1%)	Soluplus^®^ (89%)	Lutrol^®^ F68 (10%)	160/40	[40]
	Pyramid		Kollidon^®^ VA 64 (89%)		170/40	
	Hemisphere		Eudragit^®^ EPO (89%)		180/40	
	Tube		HPC (89%)		190/40	
Sustained drug release 3D-printed tablets	Cylinder	Itraconazole (35%)	HPC			[20]
			UL (65%)		170	
			SSL (65%)		170	
			SL (65%)		170	
			L (65%)		170	
	Cylinder	Tramadol hydrochloride (5%)	HPC			[26]
			SL (50%)	Mannitol (40%)	170	
			L (50%)	Magnesium stearate (5%)	170	
			HPC			
			SL (60%)	Mannitol (10%), PEO (20%), Magnesium stearate (5%)	170	
			L (60%)		170	
	Cylinder	Niclosamide (10%)	HPMC HME 15 LV (Affinisol^®^) (40.73–90%)	PEG 6000 (2.14–4.50%)	180/70	[14]
				HP- β-CD (47.13%)		
	Cylinder	Ibuprofen (20%)	PVP Kollidon^®^ 12 PF (75%)	PEG 1500 (5%)	155/25	[41]
	Ring					
	Floating device					
	Cylinder	Acetaminophen (35%)	Copovidone (56%)	Poloxamer^®^ 407 (9%)	140	[38]
	Cylinder	Metoprolol tartrate (50%)	EVA (18% VA content) (50%)		130	[34]
	Cylinder	Theophylline (40%)	Eudragit^®^ RL (0–40%)	Triethyl citrate (TEC) (12%)	80–110/40–45	[24]
			Eudragit^®^ RS (0–40%)	Glyceryl monostearate (8%)		
	Square	Acetaminophen (10–30%)	Poly (3-hydroxybutyrate) (PHB) (70–90%)		175–180	[33]
Core–shell tablets	Cylinder	Acetaminophen (40%)	Kollidon^®^ VA64 (core) (60%)		140 (core)	[8]
			Eudragit^®^ E PO (Shell) (100%)		150	
			HPMCAS LG (Shell) (30%)	PEG 4500 (Shell) (10–20%)	160	
			Eudragit^®^ RS PO (Shell) (50–100%)		110–140	
Mini-tablets	Sphere	Ritonavir/Lopinavir (25%)	HPMCAS LG (51.75%)	PEG 4000 (22.5%)	80/80	[18]
				Magnesium stearate (0.75%)		
	Cylinder	Budesonide (0.59%)	HPMC HME 15 LV (Affinisol^®^) (41.84–75.44%)	PEG 6000 (2.99–3.97%)	180/60	[15]
				H-β-CD (46.41%)		
				Adjuvant blend (citric acid,		
				tartaric acid,		
				sodium bicarbonate) (8.17–20%)		
	Cylinder	Nifedipine (25%)	HPC LF (40%)	PEG 4000 (15%)	165/70	[16]
			HPMCAS LG (19%)	Magnesium stearate (1%)		
Multi-component tablets	Cylinder Oval	Shell	Eudragit^®^ RSPO (60–90%)	Stearic acid (10–20%)	110–135	[22]
			Ethyl cellulose (15–20%)			
		Delay layer	HPC EF (90%)	PEG 400 (10%)	116	
		Delay layer	HPC EF (85%)	Glycerol (15%)	85	
		pH-responsive layer	HPMCAS LG/HG (75%)	Deionized water (12.5%)	85	
				Triacetin (12.5%)		
		Tofacinib (30%)	HPC EF (60%)	PEG 400 (10%)	114	
		Topiramate (28%)	HPC EF (52%)	PEG 400 (20%)	70	
		Metoprolol(40%)	HPC JF (40%)	PEG 400 (20%)	105	
		Levodopa/Carbidopa (32/8%)	HPC JF (35%)	PEG 400 (25%)	100	
		Clonidine (0.25%)	HPC JF (79.95%)	TEC (19.95%)	115	
		Metoprolol (25%)	Kollidon^®^ VA64 (60%)	PEG 400 (15%)	90	
		Tofacinib (40%)	Kollidon^®^ VA64 (35%)	PEG 1500 (25%)	90	
		Levodopa/carbidopa (48/12%)	PEG 8000 (36%)	Croscarmellose sodium (4%)	70	
		Topiramate (60%)	PEG 8000 (35%)	Croscarmellose sodium (5%)	75	
Orodispersible films (ODFs)	Cylinder	Clobetasol (0.2%)	PEO N10 (71.45–81.45%)	HP-β-CD (3%)	170/40	[31]
			Chitosan (15–25%)			
			HPMC HME 15 LV (Affinisol^®^)(0.35%)			
	Square	Olanzapine (5%)	PEO N-10 (70–75%)	Poloxamer^®^ 407/poloxamer^®^ 188 (5%)	160/35	[25]
			Kollidon^®^ VA64 (20%)			
Transdermal patches	Square	Ibuprofen (30%)	EVA 4030AC (70%)		80/45	[32]
		Diclofenac sodium (30%)	EVA 1821A (70%)		180/45	
Long-term implants	Ring	Paliperidone (5–10%)	PCL (90–95%)		100–110/40	[37]
	Disc					
	Cuboid	Raloxifene (10%)	PCL (45%)		90/25	[19]
	Cylinder		PEO-N80 (45%)			
	Tube					

## Data Availability

Data are contained within the article.
